# A Real-Time Wearable Assist System for Upper Extremity Throwing Action Based on Accelerometers

**DOI:** 10.3390/s20051344

**Published:** 2020-02-29

**Authors:** Kuang-Yow Lian, Wei-Hsiu Hsu, Deepak Balram, Chen-Yi Lee

**Affiliations:** Department of Electrical Engineering, National Taipei University of Technology, Taipei 10608, Taiwan; t100319009@ntut.edu.tw (W.-H.H.); t104319010@ntut.org.tw (D.B.); t104318049@ntut.org.tw (C.-Y.L.)

**Keywords:** assist system, upper extremity throwing, IMU sensor, noise filtering, intelligent action recognition, longest common subsequence

## Abstract

This paper focuses on the development of a real-time wearable assist system for upper extremity throwing action based on the accelerometers of inertial measurement unit (IMU) sensors. This real-time assist system can be utilized to the learning, rectification, and rehabilitation for the upper extremity throwing action of players in the field of baseball, where incorrect throwing phases are recognized by a delicate action analysis. The throwing action includes not only the posture characteristics of each phase, but also the transition of continuous posture movements, which is more complex when compared to general action recognition with no continuous phase change. In this work, we have considered six serial phases including wind-up, stride, arm cocking, arm acceleration, arm deceleration, and follow-through in the throwing action recognition process. The continuous movement of each phase of the throwing action is represented by a one-dimensional data sequence after the three-axial acceleration signals are processed by efficient noise filtering based on Kalman filter followed by conversion processes such as leveling and labeling techniques. The longest common subsequence (LCS) method is then used to determine the six serial phases of the throwing action by verifying the sequence data with a sample sequence. We have incorporated various intelligent action recognition functions including automatic recognition for getting ready status, starting movement, handle interrupt situation, and detailed posture transition in the proposed assist system. Moreover, a liquid crystal display (LCD) panel and mobile interface are incorporated into the developed assist system to make it more user-friendly. The real-time system provides precise comments to assist players to attain improved throwing action by analyzing their posture during throwing action. Various experiments were conducted to analyze the efficiency and practicality of the developed assist system as part of this work. We have obtained an average percentage accuracy of 95.14%, 91.42%, and 95.14%, respectively, for all the three users considered in this study. We were able to successfully recognize the throwing action with good precision and the high percentage accuracy exhibited by the proposed assist system indicates its excellent performance.

## 1. Introduction

Baseball is a popular sport played worldwide. In baseball, proper throwing technique is an important aspect for the fielding team players. Throwing action in baseball involves sequential activation of body parts through a link system and it goes from the left to right hand in a right-handed pitcher and vice versa. A coordination action of all body segments is significant to achieve a correct throwing action in baseball [1]. The involvement of swift motion during throwing action in baseball makes its analysis a challenging task. When a ball is thrown by a player, a compressive force is produced at their shoulder and elbow [2,3,4]. As a greater force is involved in throwing a ball, it is to be noted that inappropriate throwing actions often result in long-term soreness of the shoulders and elbows and even lead to serious injuries to the human body [5,6,7]. The recurrent microtrauma endured by the shoulders and elbow during the instances of powerful throwing and pitching often leads to muscular and ligament damage in overhead sports players [8,9,10,11]. Moreover, the high financial costs involved in surgical treatments of injured players and adverse effects in their throwing actions after long-term rehabilitation even if they return to sports after being fit are also areas of concern [12]. Hence, it is important to maintain correct throwing action in sports and the need for a throwing action assist system is significant. The assist system developed in this paper will be helpful for sportsmen in training sessions to learn the most basic movements of the upper arm in order to prevent improper upper extremity throwing postures to avoid the risk of getting injured. Furthermore, this assist system will be useful for injured players returning to sports after a long time to correct their throwing action and also for other players to analyze their throwing action and make further improvements in sports.

Human action analysis is considered a notable problem in different applications including physical rehabilitation, artificial intelligence, healthcare, smart living, and sports [13,14]. Various studies were carried out by researchers in the field of action recognition and movement analysis in different sports to assist the players accordingly [15,16]. For instance, Kim et al. conducted studies on detection and segmentation of sports motions using a wearable sensor [17] and estimation of knee joint forces in sport movements using wearable sensors was carried out by Stetter et al. [18]. Moreover, a wireless inertial motion-sensing system for capturing biomechanics in overhead pitching was developed by Lapinski et al. [8]. Hettiarachchi et al. developed a wearable assist system to analyze the human arm for predicting injuries due to throwing action especially in the field of cricket [19]. A wearable inertial measurement unit (IMU) for shoulder injury prevention in overhead sports was developed by Rawashdeh et al. [5]. Few action recognition studies were focused mainly on the field of baseball, and in these studies, the motion involved during pitching is evaluated. For instance, Okoroha et al. conducted a study to assess predictors of torque across the medial elbow in youth and adolescent pitchers with a mobile sensor [2] and Makhni et al. carried out a study to determine the differences in torque across pitch types and thrower demographic characteristics [20]. The relationship between elbow varus torque and arm slot and arm rotation in professional baseball pitchers was studied by Camp et al. [21]. Even though research was carried out in the field of human action recognition in sports, the need for a user-friendly and efficient assist system with advanced functionalities is significant. Hence, we have developed an efficient and user-friendly real-time wearable assist system based on the accelerometers of inertial measurement unit (IMU) sensors for upper extremity throwing action that can be easily worn on the upper arm and forearm. IMU is effectively utilized to detect movements based on acceleration, angular velocity, and rotation. A six-axis IMU composed of a three-axis accelerometer and three-axis gyroscope and a nine-axis IMU composed of an additional three-axis magnetometer are used in different fields including navigation, manufacturing, and robotics [22,23,24]. The accelerometer in IMU is utilized to measure inertial acceleration, a gyroscope is used for measuring angular rotation (pitch, roll, yaw), and a magnetometer for magnetic field measurement. It is worth mentioning that a good filtering approach is a necessity for IMU sensors as sensors’ noise and gyros drift issues can adversely impact the overall accuracy. Hence, in our developed assist system based on IMU sensors, we have included a signal pre-processing stage to achieve high accuracy.

The recognition technique incorporated in this work can be divided into three parts, namely signal measurement, signal processing and conversion, and instant identification. As we are developing a wearable device to be worn on the forearm and upper arm, it is important to design the device in a manner such that all the sensors and microcontroller circuits are integrated into the device in a miniaturized form. An embedded system is developed to measure the upper extremity throwing action signal by analyzing the acceleration signals extracted from the throwing action and the longest common subsequence (LCS) method is used for identification of the six serial phases of the throwing action. LCS is a similarity measure that was utilized effectively for string matching [25]. It can be considered a global alignment method robust to noise and outliers. The LCS method is a variation of the edit distance and its basic principle is matching two sequences by allowing them to stretch, without rearranging the sequence of the elements but allowing some elements to be unmatched [26]. It is to be noted that LCS tasks can be solved using a brute-force approach, in which all feasible subsequences of one of the strings are found followed by testing of each string to check whether it is a subsequence of other string [27,28]. Even though there is a similarity in the basic idea of LCS with that of dynamic time warping (DTW), as both lineup test and template sequence temporally based on feature distance costs, the robustness of LCS to noise and outliers make it more suitable for action recognition [29]. The throwing action assist system is developed in a user-friendly manner. Appropriate notifications and reminders allow the user to repeat the cycle to learn, rehabilitate, and correct posture and achieve the correct upper extremity throwing action.

In this paper, we have developed a real-time wearable assist system for upper extremity throwing action based on IMU sensors. Various technologies were incorporated into the system to achieve high accuracy. Moreover, this paper gives insight into the advanced action recognition process we have proposed for the development of the assist system. The rest of the paper is organized as follows: The design and architecture of the assist system are described in Section 2. Section 3 deals with the detailed throwing action analysis and various steps involved in the development of the assist system. The final results and summarization of the work are described in Section 4 and Section 5, respectively.

## 2. System Design

**Figure 1:**
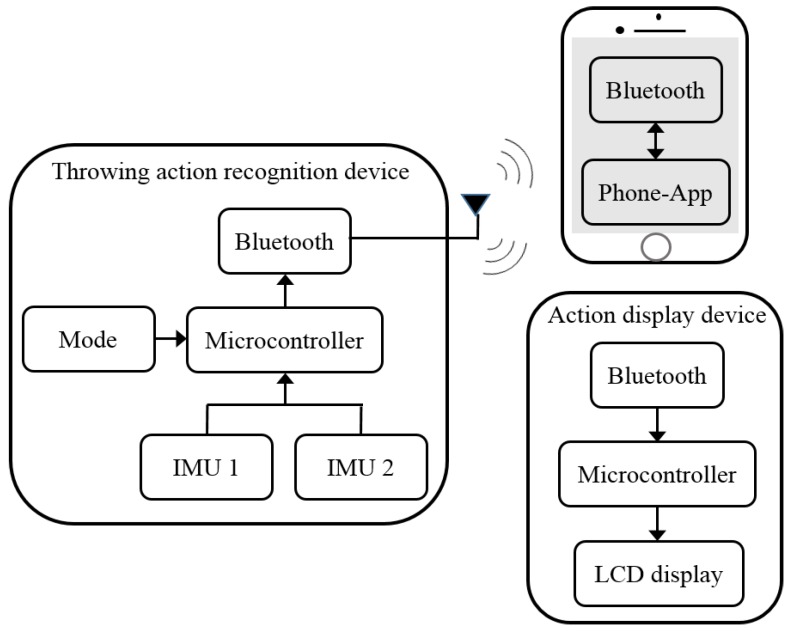
System architecture.

In this work, we have developed a real-time assist system for upper extremity throwing action and we have incorporated various hardware and software technologies as part of its development. Figure 1 depicts the hardware architecture of the developed real-time assist system. In the throwing action recognition device, the acceleration signals of the upper arm and forearm generated by throwing action are captured using two IMU sensors, respectively, as the primary step. We have used two high-precision MPU6050 inertial sensors as part of this work. The package size of MPU6050 is 4 mm × 4 mm × 0.9 mm, which is comparatively very small and also its energy consumption is very low. The IMU sensor comprises a three-axis gyroscope and a three-axis accelerometer. The function of the accelerometer of an IMU is adequate to perform action recognition in this work. For analysis, the acceleration signals captured using IMU sensors are sent to the microcontroller chip of the device. A Bluetooth module is incorporated in the action recognition device to transmit instructions to the action display device and mobile phone. Furthermore, a mode functionality is also provided in the action recognition device. Based on the analysis of the acceleration signal by the microcontroller chip of action recognition device, appropriate results are displayed on the liquid crystal display (LCD) panel of the assist system placed at the glove end. It is possible for the user to check the correctness of each phase of the throwing action at this stage by looking at the results displayed on the LCD panel. The resultant data from the microcontroller chip is further transmitted to the mobile phone interface also for the analysis of data statistics. In this way, the users are able to utilize the whole functionality of the proposed upper extremity throwing action assist system to achieve a complete system of learning, rehabilitation, and rectification.

**Figure 2:**
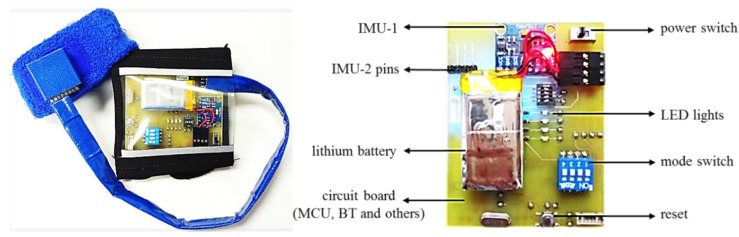
Upper extremity throwing action recognition device and the embedded circuit board.

The proposed real-time assist system for upper extremity throwing action has two modes. Mode one and mode two represent the right hand and left-hand players, respectively. As our device is a wearable one, it is designed in a user-friendly manner to help the user to use it conveniently in the real-world. Moreover, the developed assist system is an alternative to conventional motion capture biomechanical gait laboratories. The wearing design of the device includes two sections: the forearm section and the upper arm section. As shown in Figure 2, the upper extremity throwing action assist device is made as a wrist wearable type, which can be conveniently worn and taken off. Furthermore, the device is lightweight and fits smoothly to the surface of the forearm and upper arm. It is also possible to adjust the size of the device so as to fit the different arm and wrist circumferences. The proposed throwing action assist system includes a rechargeable lithium battery, a miniaturized embedded system, and a device holster. The user can replace the battery in the future if it gets drained and also easily adjust the learning mode accordingly. 

**Figure 3:**
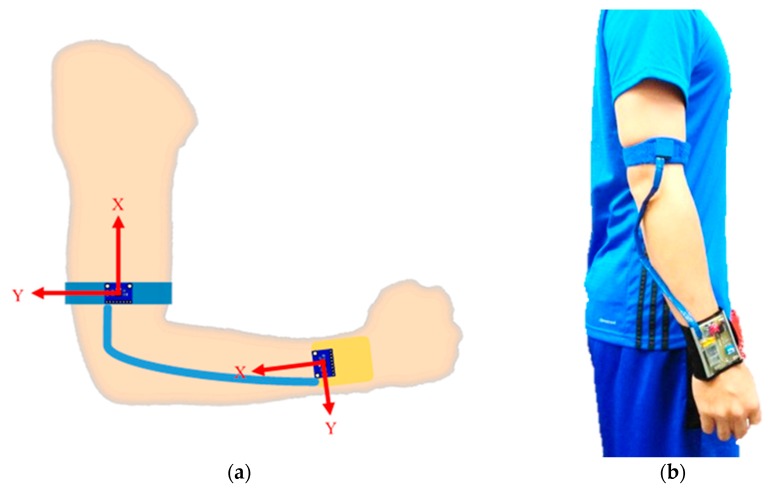
(**a**) Schematic diagram of the embedded device worn on the right arm; (**b**) and the actual diagram of the embedded device worn on the user’s arm.

**Figure 4:**
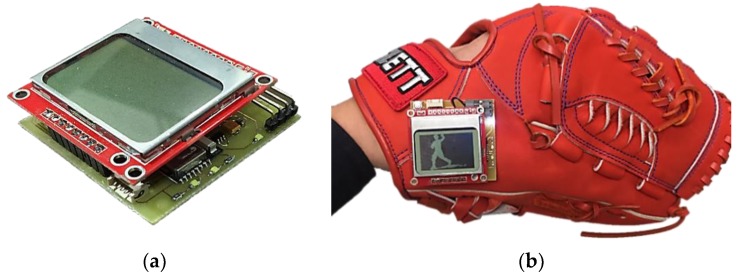
Display device of wearable assist system (**a**) LCD display device, (**b**) the device attached to the baseball glove.

We have independently designed and integrated the miniaturized embedded system with the inertial sensor acting as a spindle in order to realize the functionalities including acceleration signal measurement, wireless transmission, and data analysis of the assist system. The embedded circuit is a combination of technologies including Bluetooth wireless transmission, radio frequency (RF) wireless transmission, step-down circuits, IMU sensors, and a microprocessor. The embedded circuit board of the proposed throwing action assist system is displayed in Figure 2. We have also provided the schematic diagram of the throwing assist system worn on the right arm and also the actual diagram of the assist system worn on the user’s arm in Figure 3. The developed assist system includes a display device, which is designed for the user to instantly recognize the throwing action problems in each stage for learning, rehabilitation, and correction purposes. The display device uses a two-in-one design so that it can be attached directly to the arm or on the surface of a baseball glove. When the user needs to use the gloves for learning, rehabilitation, or correction, the display device can be directly attached to the surface of the baseball glove and can be used by turning on the switch. The function of the action display device is to display the throwing action phase in real-time and its circuitry includes an LCD display panel, Bluetooth module, step-down circuit, charging circuit, and microprocessor as shown in Figure 4.

For the design of the mobile phone user interface, we used App Inventor, which was officially opened to all users by Google at the end of 2010 [30]. The upper extremity throwing action recognition results are transmitted from the recognition device to the mobile phone through a Bluetooth wireless transmission chip. 

## 3. Throwing Action Recognition Analysis

**Figure 5:**
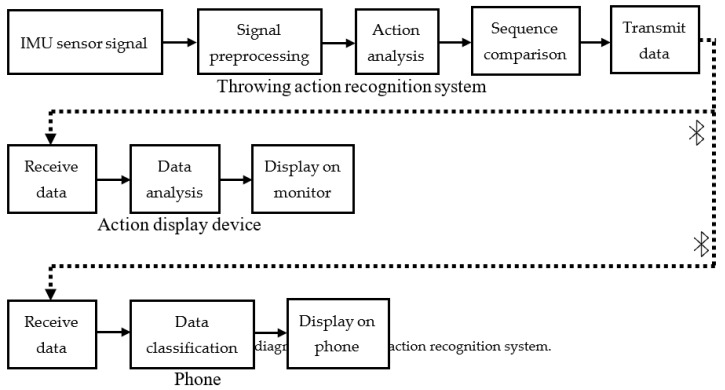
Block diagram of throwing action recognition system.

The real-time assist system for upper extremity throwing action can be used for learning, rehabilitation, and rectification purposes. Figure 5 shows the block diagram of the developed assist system. In this work, we have integrated multiple software and hardware technologies to achieve all the functionalities of the proposed assist system. The functionality of the proposed assist system can be divided into three parts as per the architecture shown in Figure 5, namely throwing action recognition, real-time throwing action data analysis, and display of statistical information. The throwing action recognition of the proposed assist system comprises different stages including signal acquisition using IMU sensors, signal pre-processing, posture interval definition, vector space conversion, sequence comparison, and throwing action identification. 

### 3.1. Signal Acquisition using IMU Sensors 

The acceleration signal is captured primarily by the two IMU sensors in the signal acquisition stage. In this study, the MPU6050 high-precision IMU sensor is used to measure the acceleration signals. The integrated circuit (IC) outputs three-axis voltage changes of acceleration via Inter-Integrated Circuit (I^2^C) communication transmission, and mainly outputs data via a serial data line (SDA) and a serial clock line (SCL). The data transmission speed can be up to 400 kHz via I^2^C. In the MPU6050, the accelerometer has built-in 16-bit analog-to-digital converter (ADC) functionality, and the 16-bit sensor output data of each axis can be represented by 2^16^, that is, 65,536 steps. The raw measurement data ranges from −32,768 to +32,768. The acceleration measurement accuracy of the forearm and upper arm is set to 2048 LSB/g and 4096 LSB/g, where LSB represents the least significant bit. The range measured by the acceleration sensor is ±16 g of acceleration signal change.

### 3.2. Signal Pre-Processing

**Figure 6:**
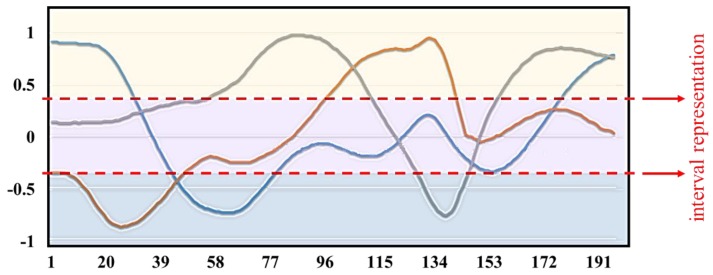
Acceleration signal after normalization procedure and its interval definition. The x-axis acceleration signal is represented by a blue line, the y-axis acceleration signal is represented by an orange line, and the z-axis acceleration signals is represented by a gray line.

It is to be noted that the acceleration signal captured by IMU sensors may be distorted due to the noise and vibrations of the sensor itself. Hence, it is important to eliminate the noise of the signal to maintain a better accuracy of the action recognition system. Therefore, a signal pre-processing stage is critical and thus we have fed the acceleration signal captured by the IMU sensors to the signal pre-processing stage. Assuming the distribution of the acceleration signal noise is Gaussian, we have chosen the Kalman filter as the best estimator [31]. When we analyze the acceleration signal captured using IMU sensors before and after applying the Kalman filter, it is evident that we were successful in eliminating noise from the signal to a greater extent. Moreover, we carried out the normalization procedure also in the signal pre-processing stage. The acceleration signal is normalized after applying the Kalman filtering technique to maintain the accuracy of the proposed action recognition system. Figure 6 shows the acceleration signal after applying the normalization procedure. Based on positive and negative values from the signal pre-processing stage, the thresholds corresponding to the system are set using the leveling technique. The threshold value for the signal triad is set to +0.35 and −0.35. The definition greater than +0.35 is set as 1, the definition between +0.35 and −0.35 is set as 0, and the definition below −0.35 is set as −1 based on the leveling technique. The forearm acceleration signal interval representation can also be visualized in Figure 6 where the yellow zone, the purple zone, and the blue zone represent the different action zones. The acceleration signal is divided into different action zones for the precise throwing action analysis. By defining different action zones based on the aforementioned threshold values we set, we could achieve better action sequence conversion based on the labeling technique which is followed in the throwing action analysis. 

### 3.3. Throwing Action Analysis 

This study considers three-quarters and overhand pitching types to analyze and identify the throwing action. Three-quarters pitching is considered the mainstream pitching method today as it possesses the common advantages of most pitching methods. When we perform three-quarters pitching, there is minimum utilization of shoulder strength and hence there will be a lower burden on the shoulder. If the elbow can be raised slightly above the shoulder line by 1 to 1.5 cm in the middle of the arm cocking, it will save energy and enhance the endurance of the pitch. The three-quarters method is the most ergonomic, which makes the movements easy to coordinate and helps to perform the action. It is the most suitable method for beginners to practice. The disadvantage is that if the elbow is lower than the shoulder when the arm is swung, it will give the elbow ligament a considerable pulling force. Therefore, it is more likely to suffer from a “baseball elbow” or laceration of the medial ligament of the elbow. The purpose of this study is to analyze the correctness of the upper extremity throwing action. By prompting the user in real-time, the user can have a knowledge of which motion phase is inappropriate and can avoid repeating it again.

#### 3.3.1. Motion Mechanics in Throwing Action Recognition 

Proper coordination of the upper and lower body movements is required to attain perfect throwing action in baseball and softball [32,33]. When we analyze the relative movement mechanics in static throwing action recognition, we can understand that it can be divided into six phases [34]. Wind-up, stride, arm cocking, arm acceleration, arm deceleration, and follow-through represent the aforementioned six phases in static throwing action recognition.

**Figure 7:**
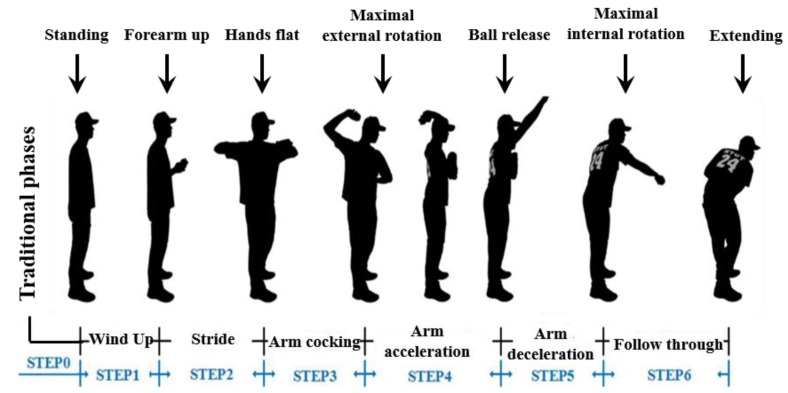
Upper extremity throwing action phases.

In this work, we have analyzed both the aforementioned static throwing action technique and Trosky’s throwing techniques. According to Trosky’s throwing drills [35,36,37], six throwing phases are introduced in the “upper-body isolation” step. The definition of the upper extremity throwing action is based on six cycles of the throwing-related motion mechanics. In this work, we have considered wind-up as Step 1, the stride period as Step 2, arm cocking as Step 3, arm acceleration defined as Step 4, arm deceleration is defined as Step 5, and follow-through is defined as Step 6. The beginning of the action is defined as Step 0, which is the automatic detection of getting ready status. The overall posture state flow of the upper extremity throwing action recognition is standing, forearm up, hands flat, maximal external rotation, ball release, maximal internal rotation, and extending. The different posture states and the six phases of upper extremity throwing action are depicted in Figure 7. 

#### 3.3.2. Action Balance Judgment 

It is necessary to establish an efficient database comprising of sample sequences of the various throwing action phases for successful action recognition. This database is necessary for the classification of throwing action phases based on the LCS algorithm. A full upper-body throwing action signal comprising the forearm and upper arm acceleration signal must be established prior to creating the throwing action database. 

**Figure 8:**
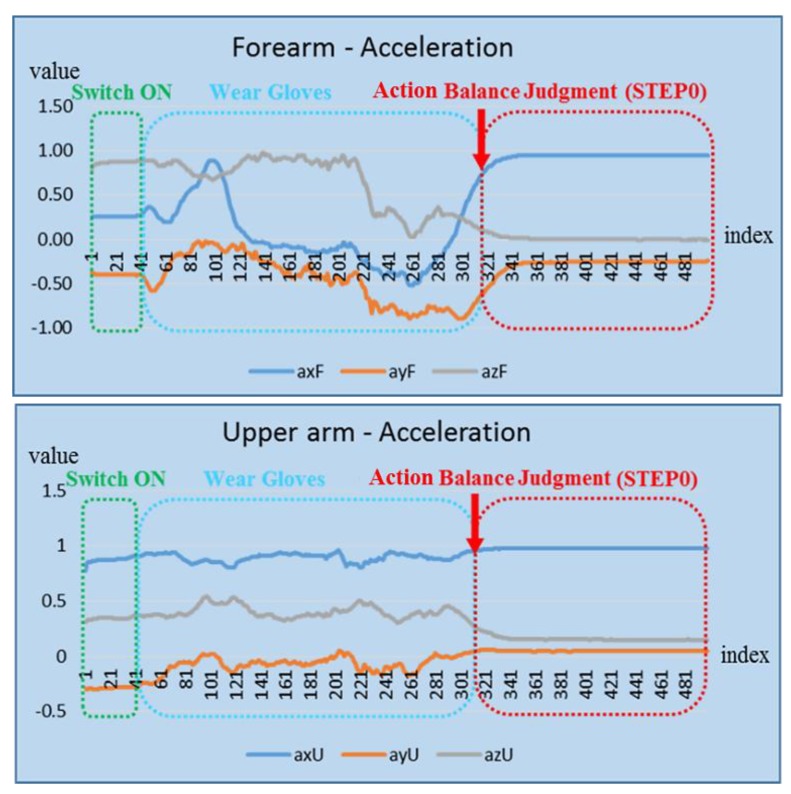
Action balance judgment for getting ready status.

Before wind-up (Step 1) of the upper-body throwing phase, the signal should be processed based on getting ready status corresponding to Step 0 in the definition of the upper arm posture phase. The main purpose of this procedure is to prevent the user from wearing the device incorrectly so that the system function will not be affected adversely. Step 0 also helps to isolate the noise signal that affects the throwing action recognition accuracy. Figure 8 depicts the action balance judgment for the getting ready status. Based on the trend of the waveforms of the forearm and upper arm defined under different action zones and with the help of signal analysis and processing, the correct posture and inappropriate posture of the forearm and upper arm can be clearly distinguished. The difference between these rules is then used to define the throwing action database sequence. 

#### 3.3.3. Throwing Action Sequence Conversion

**Figure 9:**
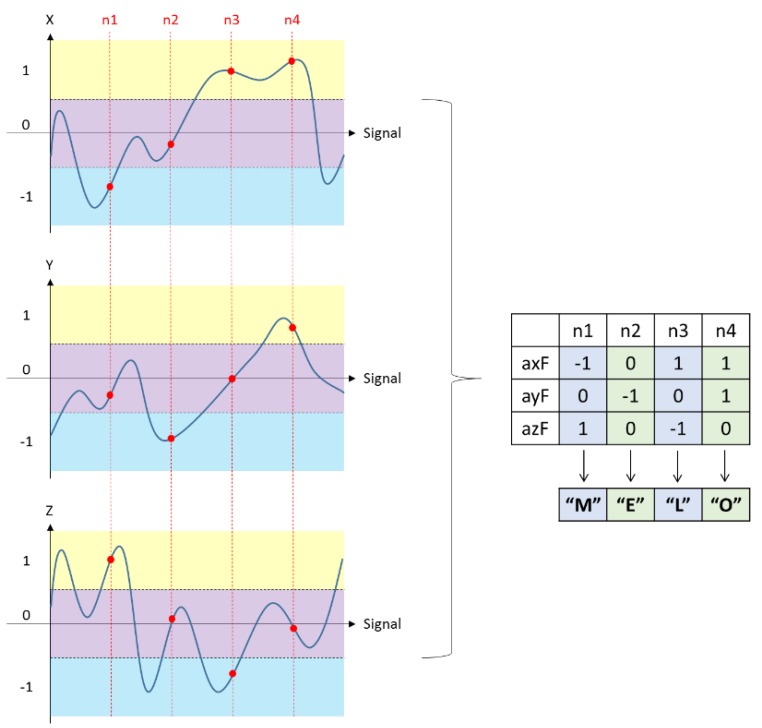
Action sequence conversion process.

After defining the different action zones, vector space representation of the signal is carried out. Based on the labeling technique followed in this system, “1” and capital letters “A–Z” are used to represent the 27 kinds of symbols quantified by the acceleration of the forearm; “2” and lower case “a–z” represents the acceleration of the upper arm. The action sequence conversion process based on the labeling technique is described in Table 1. Analyzing Table 1, we can get a clear idea of how we have used the labeling technique in this work. Moreover, we have detailed the action sequence conversion process based on the labeling technique using an apt example given in Figure 9.

Each throwing stage includes multiple action sequences, which are all converted from the signal measured by the throwing action process. Step 1 denotes the action process from standing to forearm up, which is measured by the sensor of the forearm in Step 1. The sequence is “EE⋯EEPP⋯P” and the sequence measured by the sensor on the upper arm is “pp⋯pppp⋯p”. Step 2 is the action process for forearm up to hands flat, measured by the sensor of the forearm. The sequence is “MM⋯MMYY⋯Y” and “AA⋯AAMM⋯M”, and the sequence measured by the sensor on the upper arm is “aa⋯aakk⋯k”. Similarly, the sequences of Step 3 to Step 6 are also measured. For the sake of convenience, we denote the signal sequences from Step 1 to Step 6 as follows: 

Step 1 = {E-P}⊕{p}

Step 2 = {A-M-Y}⊕{a-k}

Step 3 = {H-B-G}⊕{r-y-m}

Step 4 = {M-F-L-D}⊕{y-r}

Step 5 = {A}⊕{a}

Step 6 = {K}⊕{k}.

The sequence of the correct throwing action phases obtained from this measurement is summarized in Table 2. We have carried out continuous action recognition using sequence comparison. For a systematic comparison of the processing method, Steps 1–6 are divided into a sequence of 1–2 characters, followed by overlapping of the sequence to recognize whether the sequence is continuous. A complete sequence of actions will consist of the following sequential steps:

Step 1 = {E-P} ⊕ {p}

Steps 1–2 = {Y-E} ⊕ {k-p}

Step 2.1 = {M-Y} ⊕ {k}

Step 2.2 = {A-M} ⊕ {a-k}

Steps 2–3 = {G-A} ⊕ {m-a}

Step 3.1 = {B-G} ⊕ {y-m}

Step 3.2 = {H-B} ⊕ {r-y}

Steps 3–4 = {D-H} ⊕ {r}

Step 4.1 = {L-D} ⊕ {r}

Step 4.2 = {F-L} ⊕ {y-r}

Step 4.3 = {M-F} ⊕ {y}

Step 5 = {A-M} ⊕ {a-y}

Step 6 = {K-A} ⊕ {k-a}

Furthermore, it is to be noted that the number of characters in the sequence of throwing action is dependent on the user’s throwing speed. A slower throwing action results in a greater number of sequence characters and a faster throwing action will have a smaller number of sequence characters. For instance, when the user throws faster, the sequence of Step 1 will look like “###EEEEPPPP###” (# represents other signals). As the action takes a relatively short time, the number of characters will be less; on the contrary, when the user throws slowly, the sequence of Step 1 will be like “###EEEEEEEEEEPPPPPPPPPP###”. In this scenario, because of the slower action, there will be more identical characters.

### 3.4. Action Identification Based on LCS Algorithm 

In this work, we have used the LCS algorithm to compare different sequences and thereby analyze improper postures if any. The LCS algorithm is a sequential comparison algorithm, which is often used in different fields. Since the LCS algorithm has the advantages of high accuracy and less complexity, it is quite suitable for the instantaneous similarity verification. Consider there are two sequences, $A=\;a_{1}\ldots a_{m}\;$ and $B=\;b_{1}\ldots b_{n\;}$. The LCS length of the two sequences A and B can be obtained from the recursive relation, as shown in the following equation.

$$LCS(m,n)=\{\begin{array}{c} 0\\ LCS(m-1,n-1)+1\\ \max(LCS(m-1,n),LCS(m,n-1)) \end{array}\begin{array}{c} if\\ if\\ if \end{array}\begin{array}{c} m=0\\ m,n>0\\ m,n>0 \end{array}\begin{array}{c} or\\ \&\\ \& \end{array}\begin{array}{c} n=0\\ a_{m}=b_{n}\\ a_{m}\neq b_{n} \end{array}$$

Based on the equation, we can calculate the longest common subsequence length between two sequences, which can be used as a comparison cumulative length matrix by a dynamic two-dimensional array. According to the recursive relationship, each position in the matrix is solved and stored. The calculation process is solved from the upper left to the lower right of the matrix, which is the longest common subsequence length representing the two sequences.

**Figure 10:**
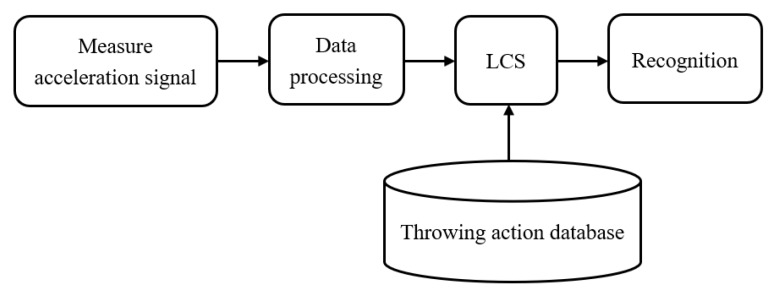
Action identification process.

Based on the action balance judgment (Step 0), the system will decide whether to perform the throwing action recognition or not. If the action balance judgment step is correct, then the sequence of each phase is compared with the continuous action. When the sequence of each action made by the user conforms to the alignment sequence in the throwing action database, the system will automatically process the LCS algorithm and carryout throwing action recognition. Contrarily, if it does not match with the alignment sequence in the throwing action database, the system will not process the LCS algorithm to carryout throwing action recognition. This method will increase the speed of recognition and recognition rate of the overall system and hence we can achieve continuous and accurate recognition of different throwing actions in real-time. Figure 10 depicts the action identification process based on the LCS algorithm. According to the process, after the comparison of the throwing posture sequence between the current arm and the upper arm is completed, the forearm and the upper arm must simultaneously meet the system to recognize that the throwing posture phase is “OK!!”. If any one of the aforementioned cases is not correct, the system will identify the error type and throw an erroneous action reminder as depicted in Table 3.

## 4. Experimental Results and Discussion

**Figure 11:**
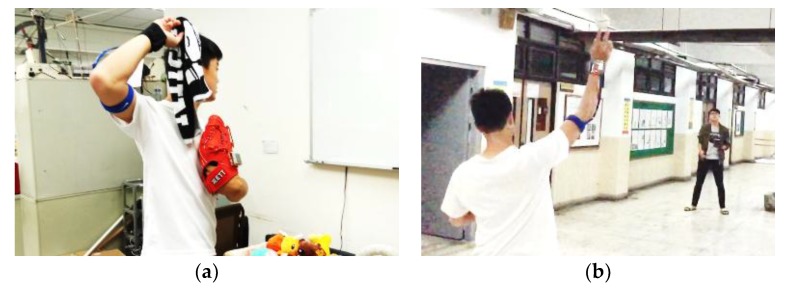
(**a**) Towel throwing method; (**b**) baseball throwing.

In order to verify the usability and authenticity of the developed wearable assist system for upper extremity throwing action, we have carried out several experiments including empty-handed simulation of the upper extremity throwing action, towel throwing method, and baseball throwing method to simulate the actual throwing posture. Figure 11 depicts the towel throwing and baseball throwing experiments we have conducted to test all kinds of upper extremity throwing actions as part of the work.

**Figure 12:**
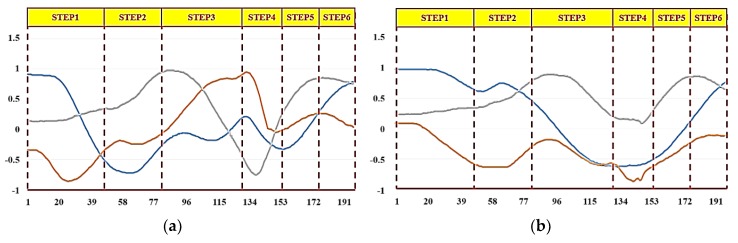
Complete action signal of the right hand: x-axis acceleration signals (blue line); y-axis acceleration signals (orange line); z-axis acceleration signals (gray line). (**a**) forearm, (**b**) upper arm.

**Figure 13:**
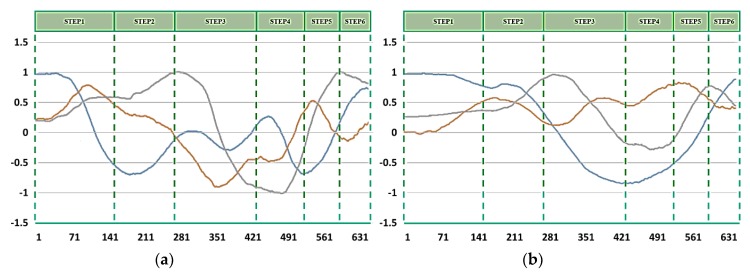
Complete action signal of the left hand: x-axis acceleration signals (blue line); y-axis acceleration signals (orange line); z-axis acceleration signals (gray line). (**a**) forearm, (**b**) upper arm.

**Figure 14:**
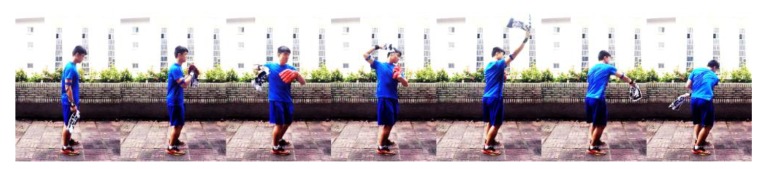
Signal measurement in continuous throwing action.

We carried out throwing experiments using both right and left hands and recorded the respective complete action signal. Figure 12 and Figure 13 show the resultant complete action signal of the forearm and upper arm of the right hand and the left hand, respectively. The photograph of the continuous action signal measurement experiment carried out in this work is taken and is presented in Figure 14. All the signals from the upper extremity throwing action can be combined to form a complete action signal and then continuous upper extremity throwing action can be identified based on the algorithm.

**Figure 15:**
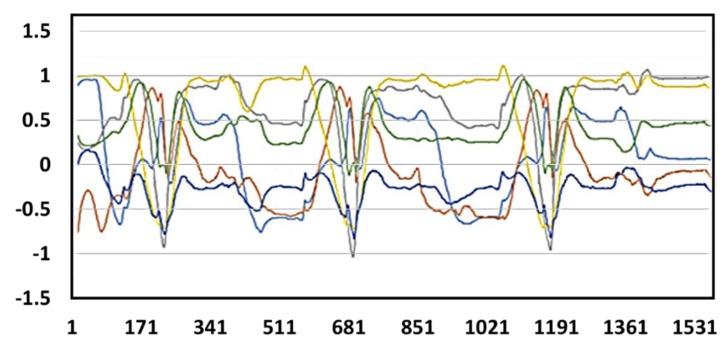
Continuous action signal in multiple throws using right hand.

**Figure 16:**
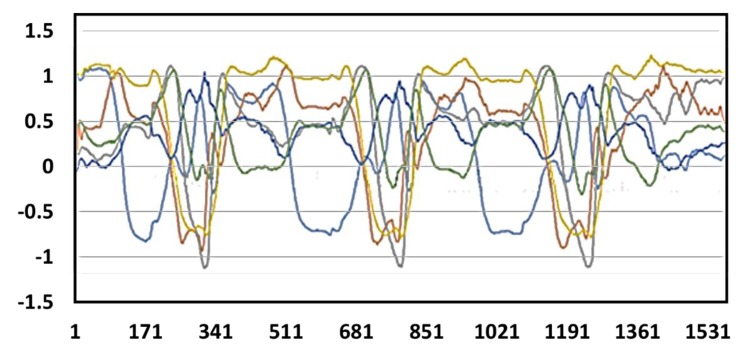
Continuous action signal in multiple throws using the left hand.

**Figure 17:**
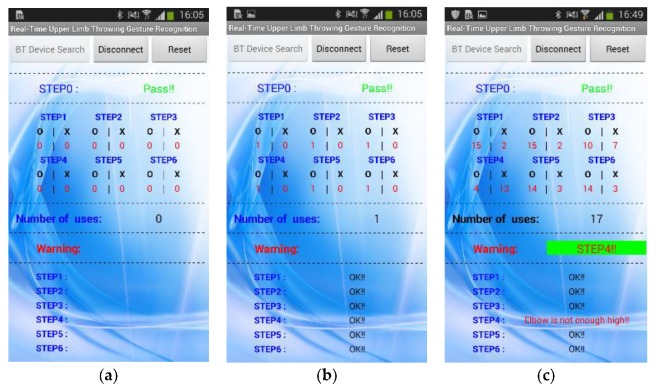
Mobile phone interface displaying: (**a**) status of Step 0; (**b**) throwing action data statistics for correct upper extremity throwing action; (**c**) throwing action data statistics for incorrect upper extremity throwing action.

We have further tested the practicality of the developed upper extremity throwing action assist system by carrying out experiments involving consecutive multiple throws using both hands. The continuous action signal during multiple throws using the right hand and the left hand are depicted in Figure 15 and Figure 16, respectively. Furthermore, the user can view the real-time upper extremity throwing action results in the display device attached at the glove end and also mobile phone. Figure 17 shows the mobile phone interface we have developed and connected to the throwing action recognition system. In an improper throwing action, the user can clearly understand in which stage he has gone wrong by checking their mobile phone and can try to correct that particular phase as shown in Figure 17. After the user completes a correct upper extremity throwing cycle, the display device will output “S1: OK!!”, “S2: OK!!”, “S3: OK!”, “S4: OK!!”, “S5: OK!!”, and “S6: OK!!”. Furthermore, an instant message will be sent to the mobile phone following the throwing action. The mobile phone message when an incomplete upper extremity throwing action was performed can be visualized in Figure 17c. In this scenario, as the elbow of the user is not high enough, the mobile phone interface has displayed the message as “STEP1: OK!!” “STEP2: OK!!”, “STEP3: OK!!”, “STEP4: Elbow is not enough high!!”, “STEP5: OK!!”, and “STEP6: OK!!”. From this experimental result, it is evident that how accurately the developed assist system recognizes the problems in throwing action.

The user can analyze the data statistics received in the mobile phone and this functionality makes this system more user-friendly and efficient. The warning message displayed in the warning area of the mobile application helps the user to understand the phase of improper action, and in this way, the user can avoid injuries due to improper action and make improvements in throwing action. With the help of the mobile phone interface connected to the system, even other people apart from the user can observe the user’s learning, rehabilitation, and rectification status. 

We have carried out a performance evaluation of the developed assist system for upper extremity throwing action. The experiment is performed with three users, where two users (User 1 and User 2) performed a right-handed throwing action and one user (User 3) performed a left-handed throwing action. Each user performed 50 throwing actions, the correct throwing action identification rate at each throwing stage was evaluated, and the respective percentage accuracy was computed. Table 4 shows the performance evaluation results we got for all three users from the experiment we have conducted. We can observe high accuracy exhibited by the assist system at every stage of the throwing action for all three users. The average percentage accuracy of all throwing stages for User 1, User 2, and User 3 are 95.14%, 91.42%, and 95.14%, respectively. So, the overall accuracy of our throwing assist system is estimated at 93.9%. Furthermore, when we analyze the table, we can infer that Step 0 has the highest identification rate with an average percentage accuracy of 99.33% and the maximal external rotation stage has the lowest accuracy of 85.33%. This high percentage accuracy indeed proves the outstanding performance of our developed upper extremity throwing action assist system. 

The need for an accurate assist system for baseball throwing was a necessity to avoid injuries and improve rehabilitation to restore lost skills and regain maximum self-sufficiency faster. We believe the development of this wearable assist system for upper extremity throwing action in baseball will be a great help for the users. The practicality of the assist system is evident from the high percentage accuracy the system exhibited during the experiment. Moreover, some of the major advantages of this developed throwing assist system include ease of use, low cost, automated analysis, instant message feedback, and high accuracy. Even though the throwing assist system developed in this work is better suited for baseball, the basic principle used in this system can be further modified to be used in other sports where throwing action is involved, particularly in cricket and softball. For instance, in the case of softball, the body movements involved during the throwing action is different from that of baseball. Hence, we have to make suitable changes to the different stages involved in this work, its definition, and also the reference data have to be replaced with appropriate data developed from throwing action in softball. In the developed wearable assist system, in case if the user does not wear the device properly in their arm, it may adversely affect the accuracy of throwing action recognition due to sensor shift. So, it is important for the user to wear the device correctly to achieve good results. This work can be further extended to make the developed throwing action recognition device robust to sensor shift. The greatest challenge in the future for the assist system is to miniaturize the embedded circuit board using application-specific integrated circuit (ASIC) technology. In this case, the system reliability can be further improved and the users can wear the small-size device on the right spot of wrist conveniently.

## 5. Conclusions

In this work, we have successfully implemented a wearable real-time upper extremity throwing action assist system based on the IMU sensor. We were able to efficiently integrate appropriate hardware at the circuit level in the development of the assist system and also miniaturize the device for convenient usage. Various intelligent action recognition functionalities are added to the system for better analysis of the results. Moreover, this work involves different software and hardware technologies, including the LCS algorithm, noise filtering based on the Kalman filter, the normalization technique, Bluetooth wireless transmission, real-time display capabilities, and hardware design. We have efficiently tested and analyzed different stages of upper extremity throwing action as part of the experiment for the development of an efficient throwing action recognition system. All the throwing stages exhibited a good identification rate for all the users considered for the experiment. The high percentage accuracy achieved for the throwing action assist system is evidence for its excellent performance and its practicality in real-time action recognition.

## Figures and Tables

**Table 1:** Action sequence conversion process based on the labeling technique.

Status	axF, axU	ayF, ayU	azF, azU	Symbol	Status	axF, axU	ayF, ayU	azF, azU	Symbol
0	0	0	1	A, a	14	1	1	0	O, o
1	0	1	0	B, b	15	1	−1	0	P, p
2	1	0	0	C, c	16	−1	1	0	Q, q
3	0	0	−1	D, d	17	−1	−1	0	R, r
4	0	−1	0	E, e	18	1	1	1	S, s
5	−1	0	0	F, f	19	1	1	−1	T, t
6	0	1	1	G, g	20	1	−1	1	U, u
7	0	1	−1	H, h	21	−1	1	1	V, v
8	0	−1	1	I, I	22	1	−1	−1	W, w
9	0	−1	−1	J, j	23	−1	1	−1	X, x
10	1	0	1	K, k	24	−1	−1	1	Y, y
11	1	0	−1	L, l	25	−1	−1	−1	Z, z
12	−1	0	1	M, m	26	0	0	0	1, 2
13	−1	0	−1	N, n					

**Table 2:** Throwing action sequence definition.

Throwing Step	Throwing Action Process	Continuous Action Sequence Definition	
		Forearm	Upper Arm
Step 0	Standing	Action balance judgment	
Step 1	Standing → forearm up	P	p
		E	
Step 2	Forearm up → hands flat	Y	k
		M	
		A	a
Step 3	Hands flat → maximal external rotation	G	m
		B	y
		H	r
Step 4	Maximal external rotation → ball release	D	r
		L	
		F	y
		M	
Step 5	Ball release → maximal internal rotation	A	a
Step 6	Maximal internal rotation → extending	K	k

**Table 3:** Erroneous action definition for different stages of throwing action.

Throwing Step	Throwing Action Process	Erroneous Action Definition
Step 0	Standing	Worn too inside or outside
Step 1	Standing → forearm up	Error-1
Step 2	Forearm up → hands flat	Error-2
		Inverted W
Step 3	Hands flat → maximal external rotation	Error-3
		Elbow is too low
		Forearm flyout
Step 4	Maximal external rotation → ball release	Error-4
		Elbow is not high enough
Step 5	Ball release → maximal internal rotation	Error-5
Step 6	Maximal internal rotation → extending	Error-6
		No follow-through

**Table 4:** Performance evaluation of throwing action recognition.

Throwing Stage	User 1		User 2		User 3	
	Identification Rate	Accuracy (%)	Identification Rate	Accuracy (%)	Identification Rate	Accuracy (%)
Step 0	50/50	100	49/50	98	50/50	100
Step 1	49/50	98	49/50	98	49/50	98
Step 2	47/50	94	45/50	90	48/50	96
Step 3	46/50	92	42/50	84	45/50	90
Step 4	44/50	88	41/50	82	43/50	86
Step 5	49/50	98	47/50	94	49/50	98
Step 6	48/50	96	47/50	94	49/50	98
